# Involvement of genetic factors in the response to a variable-dosing ranibizumab treatment regimen for age-related macular degeneration

**Published:** 2010-12-07

**Authors:** Slawomir J. Teper, Anna Nowinska, Jaroslaw Pilat, Andrzej Palucha, Edward Wylegala

**Affiliations:** 1 Department of Ophthalmology, Okregowy Szpital Kolejowy, Katowice, Poland; 2 Genomed, Warsaw, Poland

## Abstract

**Purpose:**

To determine whether gene polymorphisms of the major genetic risk factor for age-related macular susceptibility 2 (*ARMS2* A69S) and the complement factor H Y402H influence the response to a variable-dosing treatment regimen with ranibizumab for age-related macular degeneration.

**Methods:**

This prospective cohort study included 90 patients (90 eyes) with exudative age related macular degeneration (AMD) treated with ranibizumab. Patients underwent a 1-year treatment as in the Study of Ranibizumab in Patients with Subfoveal Choroidal Neovascularization Secondary to Age-Related Macular Degeneration (Mitchell et al.). Injections were administered monthly when a patient lost five letters on the Early Treatment Diabetic Retinopathy Study chart or gained 100 μm in central subfield retinal thickness (CSRT). Genotypes (rs10490924 and rs1061170) were analyzed using gene sequence analysis. Best-corrected visual acuity (BCVA) and CSRT values were compared between ARMS2 and complement factor H genotypes. Multiple regression analysis was used to assess the statistical significance.

**Results:**

Mean increase in visual acuity was 4.44±8.12 letters with a 103.63±94.7 µm decrease in CSRT. BCVA improvement was statistically significant in all genotype groups except in homozygous 69S in the *AMRS2* gene. CSRT and BCVA changes were correlated (r=0.2521; 95% CI: 0.04746–0.4364, p=0.0165). Multiple regression analysis revealed a significant impact of 69S (p=0.015) on the change in BCVA.

**Conclusions:**

Visual acuity did not improve during the study in patients homozygous for ARMS2 69S, despite a decrease in CSRT. Further investigation is needed to confirm our findings and understand the mechanisms involved.

## Introduction

The complement factor H (*CFH*) and the age-related macular susceptibility 2 (*ARMS2*) variants Y402H and A69S are major genetic risk and progression factors in age-related macular degeneration (AMD) [1]. The CFH protein is responsible for regulating the complement alternative pathway, which based on the significant number of risk-modifying single nucleotide polymorphisms (SNPs) identified among protein cascade genes in AMD, is crucially involved in the etiology of AMD [2]. The role of ARMS2 has not been fully elucidated; however, some findings suggest that it is involved in the extracellular matrix [3]. Due to strong linkage disequilibrium between *ARMS2* and HtrA serine peptidase 1 (*HTRA1*; a serine peptidase gene) and the equal contribution of their variants (rs11200638 and rs10490924) to AMD, these genes are usually mentioned together [4].

AMD remains a leading cause of legal blindness in developed countries [5]. Vascular endothelial growth factor (VEGF) inhibition via injection of anti-VEGF monoclonal antibodies (e.g., bevacizumab and ranibizumab) has become the gold standard for AMD treatment in the last decade based on findings from the MARINA and ANCHOR studies [6,7]. Questions raised about the costliness and safety of monthly intravitreal injections of ranibizumab, however, have led to the search for new prognostic factors. Increasing the period between treatments decreases the rate (0.05% per injection) of endophthalmitis, one of the most common and potentially vision damaging complications [8].

Ranibizumab inhibits all VEGF isoforms, and thus, improves the efficacy of VEGF treatment [6,7]. The VEGF 121 isoform, however, is a neurotrophic factor and the long-term effects of its inhibition are not known [8]. Different regimens have been investigated in an effort to avoid potential complications and optimize outcomes. Evidence to support the necessity of injections given every month for the first three months is related to the clear best-corrected visual acuity (BCVA) benefit during this period [6,7], but the treatment regimen following this period is a matter of debate. The Study of Ranibizumab in Patients With Subfoveal Choroidal Neovascularization Secondary to Age-Related Macular Degeneration (SUSTAIN) was one of the most important multi-centered clinical trials intended to determine if fewer, carefully timed injections could provide results similar to those in the MARINA and ANCHOR studies [9]. In addition, the cost of monthly treatment could be reduced by at least half if the SUSTAIN criteria were applied to determine the treatment regimen.

Because AMD is a complex disease with a strong genetic background, pharmacogenomics may allow for more individualized therapy.

The purpose of the present study was to determine whether gene polymorphisms affect the response to a variable-dosing regimen treatment with ranibizumab (Lucentis, Genentech/Novartis) in patients with choroidal neovascularization (CNV) subsequent to AMD. Gene variants selected for the study were *ARMS2* A69S – rs10490924 and *CFH* Y402H – rs1061170. Their putative influence on treatment efficacy was previously reported in patients undergoing photodynamic therapy (PDT) and bevacizumab treatment [10–14], and the effects of treatment with ranibizumab and *CFH* Y402H have been studied [15]. We chose variants with the highest contribution to the disease for the present study.

## Methods

A cohort of 90 consecutive patients (90 eyes; 47 women and 43 men; Caucasians; mean age, 71.62±8.4 years) of the eye clinic at the OSK Hospital in Katowice participated in the study. Active subfoveal CNV subsequent to AMD was confirmed with fluorescein angiography (FA) and optical coherence tomography (OCT) at baseline. All patients had intraretinal cysts or subretinal fluid or both in the fovea. Of the 90 patients, 74 were not treatment- naïve. Patients were enrolled in the study at least 3 months after any VEGF inhibitor injection, and 6 months after PDT or intravitreal triamcinolone. Subdividing patients into treatment groups was not possible due to the large variety of treatment modalities they had undergone before the study (PDT, ranibizumab, bevacizumab, pegaptanib, steroids, and combination therapy).

The study was a 1-center, 1-year, prospective cohort study that was performed by a genotype-masked study team comprised of BCVA and OCT technicians as well as the treating investigator. Genetic factors were not revealed to the study team until the end of the final follow-up visit. All patients provided written informed consent before any study procedure was initiated. The study was approved by the Ethics Committee of the Medical University of Silesia, Katowice, Poland (NN-6501–158/I/07) and adhered to the tenets of the Declaration of Helsinki.

In our study, patients with subfoveal CNV subsequent to AMD underwent a 12-month ranibizumab treatment. The SUSTAIN study criteria were used to determine the need for reinjection after the first three monthly injections.

Visits were scheduled every month and subjects were reinjected each time one of the following criteria was met:

– loss of 5 letters on the Early Treatment Diabetic Retinopathy Study (ETDRS) charts compared to the highest number of letters during the first 3 months of the study; and

– gain of more than 100 µm in central subfield retinal thickness (CSRT) compared to the lowest CSRT during the first 3 months of the study.

A CSRT value greater than 225 μm was a prerequisite for reinjection after the first 3 months. A dose of 0.5 mg/0.05 ml ranibizumab was used for each treatment.

### Examinations

All patients underwent a thorough examination at baseline, including BCVA, fundus photography, FA, OCT, slit-lamp examination, indirect ophthalmoscopy, and Goldmann applanation tonometry. The study visit schedule is shown in Table 1.

**Table 1:** Study visit schedule.

	**Visit**					
**Procedure**	**Baseline**	**I-III**	**IV-V**	**VI**	**VII-XI**	**XII**
BCVA (ETDRS)	+	+	+	+	+	+
Tonometry	+	+	+	+	+	+
Slit-lamp examination and indirect ophthalmoscopy	+	+	+	+	+	+
OCT	+	+	+	+	+	+
FA	+	-	-	+	-	+
Ranibizumab injection	-	+	optional	optional	optional	-

BCVA – best-corrected visual acuity, ETDRS – Early Treatment Diabetic Retinopathy Study charts, OCT – optical coherence tomography, FA – fluorescein angiography.

Visual acuity was measured at 4 m with the ETDRS charts by one of two experienced testers after standardized refraction. OCT Stratus III, software version 4.0.2 (Zeiss Meditec, Dublin, CA) was used to assess the retinal morphology (Retinal Thickness Map protocol) and CSRT (Fast Retinal Thickness Map protocol). All scans were acquired by the same experienced OCT technician. FA and fundus photography (Visucam FF450+; Zeiss) were performed and interpreted by an experienced physician (J.P.) blinded to. Initial neovascular activity, and size and type of lesion (predominantly classic, minimally classic, occult) were assessed.

### DNA collection, isolation, amplification, and sequencing

DNA was isolated from dry blood samples collected on FTA^®^ cards (Whatmann, Maidstone, UK). For DNA isolation, a disc (diameter, 2.0 mm) was punched and collected in a sterile microcentrifuge tube. DNA was isolated using the lysis and neutralization solutions from the REDExtract-N-Amp™ Blood PCR Kit (Sigma-Aldrich, St. Louis, MO) according to the manufacturer’s protocol. For PCR amplification of the *ARMS2* (5′-ATA CCC AGG ACC GAT GGT AAC-3′ and 5′-AGA GGA AGG CTG AAT TGC CTA-3′ primer pair) and the *CFH* (5′-TTG ACT AAT GCC CAT TAA TAG GAG-3′ and 5′-TTG ATA TTT CTT TTT GTG CAA ACC-3′ primer pair) allele, the 2X PCR reaction mix from the same kit was used with 1 μl of DNA sample and 5 pmol of each primer in a 25-μl reaction mix. The amplification conditions were as follows: 95 °C initial denaturation for 3 min followed by 35 cycles of 94 °C denaturation for 10 s, 58 °C (for *ARMS2*) or 50 °C (for *CFH*) annealing for 20 s, and elongation at 72 °C for 40 s. In all PCR reactions, a final elongation step was applied at 72 °C for 7 min. The quality and quantity of the PCR products was verified on 1% agarose gels by electrophoresis in Tris/borate/EDTA buffer. Approximately 20 ng of each PCR product was purified using the ExoSAP-IT^®^ enzyme mix (GE Healthcare, Buckinghamshire, UK) according to the manufacturer’s instructions and directly submitted for DNA sequence analysis.

### Statistics

Baseline differences between genotype groups were tested (i.e., BCVA, CSRT, type of lesion, area of lesion) using the Kruskal–Wallis test. BCVA and CSRT before and after treatment were compared. A Wilcoxon pair test was used to assess statistical significance. The possible relationship between baseline factors and both visual acuity and CSRT change was tested using multiple regression analysis. The following parameters were taken into consideration: age, sex, Y402H and A69S polymorphisms, CNV type, BCVA, CSRT, and lesion area at baseline. The correlation coefficient for BCVA and CSRT changes was calculated. MedCalc 10.2.0.0 (MedCalc Software bvba, Mariakerke, Belgium) was used for all analyses.

## Results

### BCVA and CSRT

The average increase in visual acuity (4.44±8.12 letters) was lower than that reported in the MARINA and ANCHOR studies, with a 103.63±94.7 decrease in CSRT. CSRT and BCVA changes were correlated (r=0.2521; 95% CI: 0.04746–0.4364, p=0.0165). We did not observe significant BCVA improvement in the *ARMS2* 69S homozygous group. In patients homozygous for *CFH* 402H, the significance of the BCVA change (p=0.04) was lower than in the Y402H heterozygous and 402Y homozygous groups (Table 2).

**Table 2:** Study population characteristics.

		***CFH* Y402H**		***ARMS2* A69S**			
**Genotype**	**All**	**CC**	**CT**	**TT**	**TT**	**GT**	**GG**
Genotype frequency	-	28 (31.1%)	47 (52.2%)	15 (16.7%)	26 (28.9%)	33 (36.7%)	31 (34.4%)
Lesion area	15.64	17.82	14.75	14.38	15.31	16.81	14.68
[mm^2^]	±10.63	±13.22	±10.26	±7.16	±11.88	±10.0	±10.4
Kruskal–Wallis test H (2, n=90)=0.6011253 p=0.74 H (2, n=90)=1.366674 p=0.505							
Type of CNV	31:30	11:10:7	14:16:17	6:4:5	12:9:13	12:10:11	7:11:5
pdc:mc:oc	29						
Kruskal–Wallis test H (2, n=90)=0.6248223 p=0.732 H (2, n=90)=4.495667 p=0.106							
BCVA	41.79	40.28	43.51	39.2	41.31	38.79	45.39
[letters]	±14.52	±15.62	±14.19	±13.66	±16.47	±13.7	±13.26
Kruskal–Wallis test H (2, n=90)=1.164551 p=0.559 H (2, n=90)=3.937457 p=0.14							
BCVA after 12 months	46.23	42.32	49.06	44.67	42.65	43.12	52.55
[letters]	±16.54	±16.77	±15.78	±17.82	±17.09	±16.93	±14.13
	(4.44±8.12)	(2.04±6.01)	(5.55	(5.47	(1.35	(4.33	(7.16
			±9.53)	±5.85)	±8.87)	±8.92)	±5.39)
	**p<0.01**	**p=0.04**	**p<0.01**	**p<0.01**	p=0.5	**p<0.01**	**p<0.01**
CSRT [µm]	331.95±99.05	333.71±85.53	332.85	325.87	312.85	338.18	341.35
			±100.22	±123.31	±103.54	±89.69	±105.55
Kruskal–Wallis test H (2, n=90)=1.164551 p=0.559 H (2, n=90)=3.937457 p=0.14							
CSRT after 12 months [µm]	228.32	234.61	231.36	207.07	223.58	206.09	255.97
	±72.27	±80.08	±71.59	±58.35	±82.03	±46.69	±78.80
	(103.63±94.7)	(99.11	(101.49	(118.8	(89.27	(132.09	(85.39 ±91.98)
	**p<0.01**	±83.49)	±92.87)	±122.19)	±100.66)	±88.16)	**p<0.01**
		**p<0.01**	**p<0.01**	**p<0.01**	**p<0.01**	**p<0.01**	
Injections	5.77±1.51	5.96±1.64	5.83±1.34	5.2±1.74	5.85±1.51	5.79±1.56	5.68±1.51

BCVA – best-corrected visual acuity, CNV – choroidal neovascularization (pdc-predominantly classic, mc-minimally classic, oc-occult), CSRT – central subfield retinal thickness

### Genotyping

Genotyping results and baseline characteristics of the study cohort are presented in Table 2. Genotype frequencies of both polymorphisms were in Hardy–Weinberg equilibrium.

### Treatment efficacy factors

A multiple regression analysis was performed to assess the independency of the SNP as a factor associated with treatment efficacy. The BCVA, CSRT, and number of injections are presented in Table 3, and the final BCVA, CSRT, and number of injections divided by genotype group are presented in Table 2. Marked values are statistically significant compared with the baseline value. The multiple regression analysis results are presented in Table 3 (BCVA, OCT, number of injections); this analysis revealed a significant influence of 69S homozygosity on the treatment efficacy measured with BCVA change (p=0.015).

**Table 3:** Multiple regression results.

**Dependent**	**BCVA change (final BCVA – BCVA at baseline)**				**CSRT change (CSRT at baseline – final CSRT)**				**Number of injections**			
Coefficient of determination R^2^	0.2004				0.5718				0.1122			
R^2^-adjusted	0.1215				0.5295				0.02454			
Multiple correlation coefficient	0.4477				0.7562				0.335			
Residual standard deviation	7.6097				64.9625				1.4957			
**Regression equation**	Coefficient	Std. Error	t	p	Coefficient	Std. Error	t	p	Coefficient	Std. Error	t	p
69S	−2.655	1.0654	−2.492	**0.0147**	9.486	9.0955	1.043	0.3001	0.1863	0.2094	0.89	0.3763
402H	−1.7727	1.2004	−1.477	0.1436	−10.0345	10.2473	−0.979	0.3304	0.325	0.2359	1.377	0.1722
CNV type	−1.4099	1.0405	−1.355	0.1792	−12.274	8.8828	−1.382	0.1708	0.2841	0.2045	1.389	0.1687
Lesion area at baseline	−0.114	0.07928	−1.439	0.1541	−1.2062	0.6768	−1.782	0.0784	0.01053	0.01558	0.676	0.5012
CSRT at baseline	0.01698	0.008583	1.978	0.0513	0.7378	0.07327	10.07	**<0.0001**	0.003203	0.001687	1.899	0.0611
BCVA at baseline	−0.01153	0.05763	−0.2	0.8419	−0.4074	0.492	−0.828	0.41	0.005864	0.01133	0.518	0.6061
Age	−0.1456	0.09868	−1.475	0.1441	0.7436	0.8425	0.883	0.38	0.006667	0.0194	0.344	0.7319
Gender	−0.6197	1.6361	−0.379	0.7059	9.5343	13.9674	0.683	0.4968	0.1664	0.3216	0.517	0.6063
**Analysis of variance**	DF	Sum of Squares		Mean Square	DF	Sum of Squares		Mean Square	DF	Sum of Squares	Mean Square	
Regression	8	1175.763		146.9703	8	456406.7		57050.84	8	22.9046	2.8631	
Residual	81	4690.46		57.9069	81	341830.2		4220.126	81	181.1954	2.237	
F-ratio	2.538				13.51875				1.2799			
Significance level	p=0.016				p<0.001				p=0.266			

BCVA – best-corrected visual acuity, CSRT – central subfield retinal thickness

### R38X

Sequence analysis of several *ARMS2* loci in our samples revealed the presence of an additional Arg38Opal (stop) C/T genotype (R38X). The R38X *ARMS2* SNP was present in 16 patients. In seven patients, it coexisted with A69S SNP on another allele. R38X did not correlate with the final BCVA or CSRT.

## Discussion

This prospective study revealed a correlation between the *ARMS2* genotype and ranibizumab efficacy when injected according to the SUSTAIN study protocol. Patients homozygous for the 69S variant showed a poor response, especially with regard to BCVA; this group was the only group of patients that did not gain letters on the ETDRS chart. As the function of ARMS2 remains unknown, there is currently no pathophysiological explanation for the influence of A69S. Although there were no significant effects of *CFH* Y402H, BCVA improvement was relatively low in the homozygous 402H group (p=0.04). Because the ranibizumab treatment regimen was not consistently administered to any of the subjects, the effect of the chosen reinjection criteria cannot be excluded as an important environmental factor and possible bias. In a previous, retrospective, 9-month ranibizumab study, patients were treated at the physician’s discretion [15], which is why the results are not comparable.

The first pharmacogenetic paper on AMD was published in 2007, in which no significant effects were detected in a study of 88 patients who had undergone PDT due to AMD [10]. Subsequent papers reported conflicting results. Goverdhan et al. published a study concluding that 402H may predispose to predominantly classic lesions, and patients homozygous for 402H (CC) had significantly worse results after PDT [13]. However, there were only two TT patients (homozygous for 402Y) that were studied. Furthermore, we know that patients with this type of CNV tend to respond better to PDT [16]. Brantley et al. [11] reported that if patients with predominantly classic lesions are subdivided by genotype, CC and CT patients have significantly better results. Interestingly, the CC genotype was a negative prognostic factor in a cohort treated with bevacizumab every 6 weeks [14]. This effect was also observed in a population treated with ranibizumab [15]. In our study, patients homozygous for 402H tended to have worse results, but this effect was not significant. In the previously mentioned study of treatment with ranibizumab, the *CFH* genotype seemed to influence the number of injections [15]; however, in our cohort, this effect was not significant, which might be related to the different retreatment criteria. The present study is the first to show that the 69S ARMS2 variant in homozygous subjects affects the response to ranibizumab. Despite the reduction in CSRT, BCVA showed no improvement. Thus, ranibizumab is effective for reducing macular edema, but the lack of BCVA improvement might be related to structural changes in the retina, retinal pigment epithelium atrophy, and the loss of photoreceptors.

The influence of the gene variants may have been altered if more aggressive criteria were chosen for determining treatment frequency.

Despite the significant findings in our study, a false positive association may be as likely, or even more likely than a true positive when investigating such common alleles [17]. Further studies are needed to validate these results.

### Study limitations

1. Most patients were not treatment-naïve and had undergone many different treatment modalities before the beginning of the study.

2. The number of patients was relatively low.

3. False positive associations may be common in such studies.

The genetic contribution to the variable outcomes in wet AMD treatment is likely related to many loci. The combined effects of different variants and gene–environment interactions make it difficult to detect stronger associations. Single genotypes are likely to explain only a small proportion of efficacy variation. It is also possible that risk genotypes only predispose to the development of late stages of AMD, but do not influence how these late stages progress or respond to treatment. Large samples and genome-wide analyses rather than a candidate gene approach might improve the replication of genetic associations leading to the generation of multivariate predictive models and personalized therapy. AMD has a complicated etiology, and therefore, lifestyle factors and antioxidant intake should be included in future multi-centered clinical trials.
